# Intramedullary spinal epidermoid cyst of the cervicodorsal region: A rare entity

**DOI:** 10.4103/1817-1745.66675

**Published:** 2010

**Authors:** Ashok Kumar, Pritish Singh, Pramod Jain, C.M. Badole

**Affiliations:** Department of Orthopedics and Traumatology, M.G.I.M.S., Sewagram - 442 102 Wardha, Maharashtra, India

**Keywords:** Intramedullary epidermoid cyst, meningitis, spinal dysraphism

## Abstract

Intramedullary spinal epidermoid cysts are rare, with only few cases having been reported in the literature. We are reporting a case of a 10-year-old female child who presented with symptoms of meningitis with progressive paraparesis. Magnetic resonance imaging of the spine revealed an intramedullary epidermoid cyst from C6 to D5. Near-total excision of the tumor was performed. Histopathological report confirmed the diagnosis of epidermoid cyst. The patient showed progressive recovery.

## Introduction

Intraspinal epidermoid cyst is a rare tumor. The incidence of intraspinal epidermoid cysts in children is 3% and in adults is 1%.[1–3] A large portion of epidermoid cysts are subdural and extramedullary. True intramedullary epidermoid cysts are uncommon, with <60 cases having been reported in the literature since the first reporting of the entity by Chiari in 1833. Of these, a very few have detailed radiographic evaluation. Intramedullary epidermoid cyst is common in the dorsal and lumbosacral region. Regions with two frequent localizations are T4–T6 and T11–T12, while only three cases have been reported with cervical cord involvement.[2,4,5] We report the case of a 13-year-old female patient with an intramedullary epidermoid cyst in the cervicodorsal region, which was evaluated by magnetic resonance imaging (MRI).

## Case Report

A 10-year-old female child was referred by a private practioner with history of high-grade fever on and off, pain in the nape of the neck and nuchal rigidity in combination with progressive spastic quadriparesis of 15 days duration. This constellation of symptoms pointed toward provisional etiology of meningitis or meningoencephalitis. Keeping a high probability of meningoencephalitis from this constellation of symptoms, the patient was initially evaluated and treated by pediatric side. The fever subsided with the conservative treatment. She did not have any cutaneous spine manifestation or any procedure on the spine in the past. She had a spastic paraparetic gait with power in both lower limb 4/5 and in upper limb 3/5 on the MRC scale. There was hypertonia in all four limbs, with all deep tendon reflexes exaggerated and symmetrical. Planters were extensor type. There was no sensory deficit except that the patient had occasional radiculitis of the bilateral upper limb. Sacral dermatomal sensations and bladder function was equally preserved.

**Figure 1:**
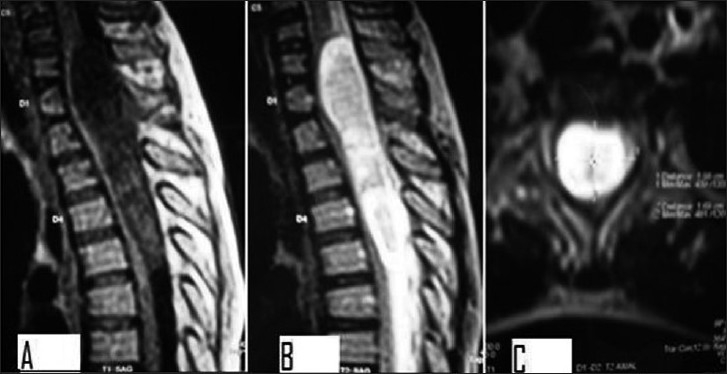
T1-weighted and T2-weighted Magnetic resonance image of the epidermoid cyst with cervico-dorsal extension, the lesion being hypointense on T1 sequence (A) and hyperintense on T2 sequence (B, C)

Initially, cerebrospinal fluid examination by gram staining, cytology and culture excluded an anticipated infective etiology. Plain X-rays of the spine were normal and not conclusive to final diagnosis. MRI revealed a well-defined nonenhancing intradural intramedullary oblong and oval cystic lesion extending from the lower border of C6 to the lower border of D5 vertebra with signal characteristics suggestive of intradural intramedullary epidermoid cyst, the lesion being hypointense on T1-weighted sequence and hyperintense on T2 sequence. The margins of the lesion had a shaggy appearance. The study did not show any spinal dysraphism [Figure 1].

Tumor was approached by laminectomy from C7 to D5. There was a distinct bulge of the dura, which, after opening, revealed a widened cord with a bulge. A midline myelotomy was performed and a pearly white lesion within the substance of the cord was seen. The lesion expressed a yellowish-white pultaceous material on incision. Near-complete dissection of cyst was performed.

**Figure 2:**
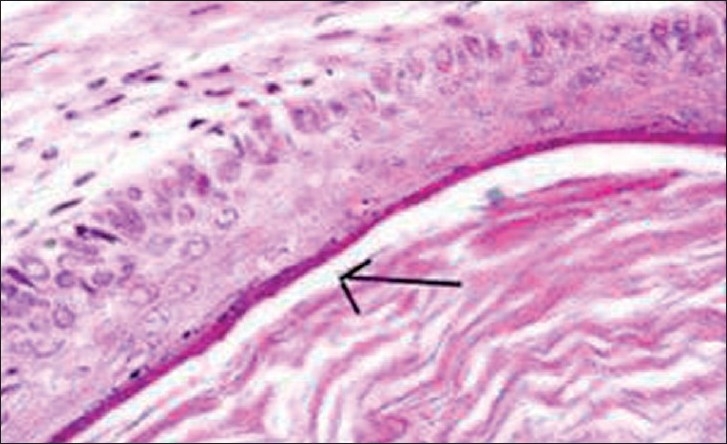
Histopathological findings of fibrous tissue lined in combination with compressed stratified squamous epithelium (arrow), conclusive of epidermoid cyst, (H & E, 40×)

Histopathological examination revealed fibrous tissue lined in combination with compressed stratified squamous epithelium. These findings were consistent with epidermoid cyst [Figure 2].

The patient made an uneventful recovery with subsequent improvement in her neurological status. The patient was provided with SOMI brace for 2 months. At the end of 2 months, the pt gained 4+/5 power in lower limb and 4/5 in the upper limb, but some residual spasticity remained.

## Discussion

Epidermoid cysts are mainly congenital as they take origin from anomalous inclusion of the ectoderm tissue during the closure of the neural tube in early fetal life and possibly may be associated with defective closure of the dural tube. This may have manifestations of other forms of dysraphism, such as syringomyelia, dorsal dermal sinus, spina bifida and hemivertebrae.[6,7] Iatrogenic penetration of the skin fragments after single or multiple spinal lumber punctures or after meningomyelocele repair may result in an acquired form of epidermoid cyst. This has been reported even years after the spine procedure.

Epidermoid tumors rarely occur in the central nervous system, and are even rarer in the spinal canal. Even the largest series of neural tumor found an incidence of spinal epidermoid cyst at 0.7%. Intramedullary localization is extremely rare. In 1989, Roux *et al*. found 47 cases of spinal intramedullary epidermoid cysts in the literature and in their clinic. In our literature survey, we have found 19 cases since then.[1,2,4–9] The commonest site of involvement is the thoracic spine usually at D 4–6 and D11–12 regions. The next site of predilection is the lumber region. Cervical cord localization is a rare occurrence, with only three cases having been narrated in the literature, as is our case.[2,4,5]

There are other entities that may pose a challenge to preoperative diagnosis, like dermoid cyst, teratoma, ependymomas, astrocytomas and hemangioma, owing to their intramedulary localization. However, typical signal intensity, absence of peritumoural edema, sharp boundaries and minimal peripheral enhancement with gadolinium confine the diagnosis to an epidermoid cyst.[4,10] Our case showed similar features to conclude a diagnosis.

Histologically, epidermoid cysts are lined by the stratified squamous epithelium supported by an outer layer of collagenous tissue. Desquamation of keratin from the epithelial lining toward the interior of the cyst produces a soft white material. Absence of skin adnexa will rule out the diagnosis of dermoid cyst.[8]

The treatment of epidermoid cyst is essentially surgical. Literature shows radiotherapy as a modality in only one case.[11] Emptying of the cyst material and removal of the capsule is the treatment of choice.[5,8,12] In our case, the capsule was not so adherent to the neural tissue and it was removed near completely without damaging the neural tissue. Attempts to completely dissect out remnants of the capsule may result in avoidable sequelae of neurodeficit. The benign nature of the epidermoid cysts offers an opportunity for a better neurological outcome if detected early.
